# 气相色谱-串联质谱法测定化妆品中15种防腐剂类过敏原物质

**DOI:** 10.3724/SP.J.1123.2023.04010

**Published:** 2024-01-08

**Authors:** Weina HAN, Tongtong LIU, Lulu WANG, Xiaoyu HOU, Jin CAO

**Affiliations:** 1.国家市场监督管理总局食品质量与安全重点实验室, 中国食品药品检定研究院, 北京 100050; 1. Key Laboratory of Food Quality and Safety, State Administration for Market Regulation, National Institutes for Food and Drug Control, Beijing 100050, China; 2.中国药科大学, 江苏 南京 210009; 2. China Pharmaceutical University, Nanjing 210009, China

**Keywords:** 气相色谱-串联质谱, 多反应监测模式, 保留指数, 防腐剂类过敏原物质, 化妆品, gas chromatography-tandem mass spectrometry (GC-MS/MS), multiple reaction monitoring (MRM), retention index (RI), preservative allergens, cosmetics

## Abstract

防腐剂成分大多具有刺激性,容易造成皮肤过敏,国内外对其在化妆品中的使用有明确的禁、限量规定。研究通过气相色谱-串联质谱(GC-MS/MS)同时测定化妆品中15种防腐剂类过敏原物质的含量,建立了基于“两柱保留指数、一个质谱匹配度”的精确鉴定方法,通过外标法定量。样品经过乙腈涡旋超声提取,无水硫酸镁除水后,分别采用DB-5MS和DB-WAX色谱柱分离检测,以电子轰击离子(EI)源为离子源,通过多反应监测模式(MRM)采集数据信息,使用保留指数对15种防腐剂类过敏原物质进行定性。经两次检测,15种防腐剂类过敏原物质在DB-5MS色谱柱上方法的检出限为0.02~0.2 mg/kg, 12种防腐剂类过敏原物质在DB-WAX色谱柱上方法的检出限为0.01~20 mg/kg。防腐剂类过敏原物质在DB-5MS和DB-WAX色谱柱上均在一定范围内线性关系良好,相关系数均大于0.99。以水、乳、面膜、膏霜为代表基质,在低、中、高3个加标水平下,15种防腐剂过敏原物质在DB-5MS和DB-WAX色谱柱上的回收率在70.1%~129.8%范围内,相对标准偏差(RSD)均小于15%(*n*=6)。采用建立的方法对80批实际样品进行检测,在DB-5MS和DB-WAX色谱柱条件下,有两批样品中均检测出禁用防腐剂4-羟基苯甲酸异丙酯;DB-5MS色谱柱条件下,有11种限用防腐剂被检出,DB-WAX色谱柱条件下,10种限用防腐剂被检出。检测结果表明:该方法使用双柱系统,准确性高,能有效避免假阳性和假阴性的检测结果,可用于化妆品中15种防腐剂类过敏原物质的检测,使用保留指数对15种防腐剂类过敏原物质进行定性,为非定向筛查提供了思路,满足监管需求。

## Abstract (en)

Most preservatives are irritating and can easily induce skin sensitivities. Therefore, both domestic and international regulations impose clear restrictions on the use of preservatives in cosmetics. Herein, gas chromatography-tandem mass spectrometry (GC-MS/MS) was employed to simultaneously analyze the levels of 15 preservative allergens in cosmetics. Further, a precise identification approach based on a two-column retention index and mass spectrometry matching degree was developed. Cosmetic samples were extracted via acetonitrile vortex ultrasound extraction and then dehydrated with anhydrous MgSO_4_. The preservative allergens were separated on two columns, namely, DB-5MS and DB-WAX. Targets were identified using electron impact ionization (EI) source and the multiple reaction monitoring (MRM) mode and characterized using a retention index calibrated by a series of *n*-alkane standards. Following two tests, the LODs for the 15 preservative allergens on the DB-5MS column were in the range of 0.02-0.2 mg/kg, while those for 12 preservative allergens on the DB-WAX column were in the range of 0.01-20 mg/kg. The preservative allergens on the DB-5MS and DB-WAX columns demonstrated strong correlations, with all correlation coefficients exceeding 0.99. The recoveries for the 15 preservative allergens were in the range of 70.1%-129.8% at low, medium, and high levels, and the relative standard deviations (RSDs) were all below 15% (*n*=6) when using water, lotion, facial mask, and cream as the representative matrix. Next, 80 batches of genuine samples were tested using the established method. Isopropyl 4-hydroxybenzoate, a prohibited preservative, was detected in two sample batches using the DB-5MS and DB-WAX columns. Additionally, 11 and 10 restricted preservative allergens were identified on the DB-5MS and DB-WAX columns, respectively. The test results indicate that the double-column system approach offers excellent accuracy, effectively preventing false-positive and false-negative results, and can detect the 15 preservative allergens in cosmetics. The use of the retention index for the qualitative detection of these preservative allergens offers valuable options for non-targeted screening and meeting regulatory criteria.

化妆品中使用防腐剂可以有效降低和控制微生物生长,减少由于微生物污染而引起的产品变质风险,避免因外源性生物污染给人体健康带来潜在危害。但大多数防腐剂都具有刺激性,容易造成皮肤过敏^[1⇓-3]^。目前化妆品中常见的防腐剂包括4-羟基苯甲酸酯、异噻唑啉酮、甲醛及其释放剂等^[4]^。其中,化妆品中异噻唑啉酮类防腐剂的使用导致部分消费者发生了严重的化妆品过敏^[5,6]^。甲醛及其释放剂导致的化妆品接触性过敏现象也十分常见^[7,8]^,因此,我国2021年修订《化妆品禁用原料目录》中明确指出化妆品中禁止使用甲醛^[9]^。而苯甲酸、4-羟基苯甲酸酯类防腐剂则相对安全,造成的过敏现象较为少见^[5]^,但也有数据表明近年来台湾地区4-羟基苯甲酸酯类接触性过敏的患病率呈上升趋势^[10]^。

为保障消费者权益和健康,各国对化妆品中允许添加的防腐剂种类、使用范围和最大允许使用浓度都做了规定。如:欧盟委员会限制化妆品中使用4-羟基苯甲酸异丙酯等5种4-羟基苯甲酸酯类物质^[11]^,并禁止免洗类产品中使用甲基异噻唑啉酮,在淋洗类化妆品中允许使用的最大限量为0.01%^[12]^。我国《化妆品安全技术规范》(2015年版,以下简称《技术规范》)也对化妆品中4-羟基苯甲酸酯、苯甲酸及其盐和酯类等防腐剂做了禁、限量规定^[13]^。对化妆品中防腐剂的使用情况进行检测和监管可以在确保产品质量、延长产品保质期的同时,尽可能保证消费者使用的安全性。

目前化妆品中防腐剂的检测主要有高效液相色谱法(HPLC)^[14⇓-16]^、液相色谱-质谱法(LC-MS)^[17⇓-19]^、气相色谱法(GC)^[20,21]^和气相色谱-质谱法(GC-MS)^[22]^,其中,GC-MS分离效能高,在化妆品防腐剂检测中已有应用,如王任等^[23]^利用HLB小柱对样品进行有效净化,然后结合GC-MS/MS测定了化妆品中8种防腐剂类成分;吕稳等^[24]^利用GC-MS技术同时检测了婴幼儿化妆品中16种防腐剂。目前利用GC-MS技术进行检测时,研究人员大多选用单色谱柱展开,但实际应用时单一柱系统可能无法满足应用需求。我国《技术规范》中对防腐剂的检测通常采用液相色谱法和气相色谱法,但其中所列出的测定方法存在检测对象不够全面、定性和定量准确性欠缺的问题,不能满足近几年国家风险监测防腐剂测定和监管的需要。实际测定过程中可能由于配方添加量低于方法检出限或方法专属性差,无法排除基质干扰等原因,易造成误判,导致防腐剂种类、含量与配方标签不符。同时对于化妆品中防腐剂类成分非定向筛查时可能存在保留时间随实验条件更改而变化、定性不够准确等问题。因此建立更灵敏、更广泛、高通量的检测手段用于化妆品安全监管十分必要。

目前分离检测多种组分的研究,大多对比多种不同色谱柱的分离情况,最终选择单根最优色谱柱开展。本文利用气相色谱-串联质谱仪,建立了采用DB-5MS和DB-WAX色谱柱检测化妆品中15种常用防腐剂的分析方法,采用正构烷烃进行保留指数(RI)校准定性,建立了基于“两柱保留指数、一个质谱匹配度”的精确鉴定方法。该方法准确性强,灵敏度高,相对全面,扩大了防腐剂检测的应用范围,既可用于非定向筛查,也可用于定量研究。选用极性相差较大的DB-5MS和DB-WAX色谱柱,可覆盖相对全面的极性范围,也可以进一步满足实际应用时需要同时检测15种防腐剂类过敏原物质外其他组分的需求。

## 1 实验部分

### 1.1 仪器与试剂

气相色谱-串联质谱仪GCMS-TQ8050(日本Shimadzu公司); DB-5MS色谱柱、DB-WAX色谱柱(30 m×0.25 mm×0.25 μm,美国Agilent公司); XP205分析天平、AL204分析天平(瑞士Mettler Toledo公司);涡旋混合器(海门市Kylin-Bell公司);超声波清洗器(北京科玺世纪科技有限公司);离心机(安徽中科中佳科学仪器有限公司)。

苯甲醇、苯甲酸、甲基异噻唑啉酮、苯甲酸异丙酯、5-溴-5-硝基-1,3-二噁烷、甲基氯异噻唑啉酮、4-羟基苯甲酸甲酯、4-羟基苯甲酸乙酯、4-羟基苯甲酸异丙酯、4-羟基苯甲酸丙酯、苯甲酸苯酯、4-羟基苯甲酸异丁酯、4-羟基苯甲酸丁酯、4-羟基苯甲酸苯酯、4-羟基苯甲酸苄酯等15种防腐剂类过敏原物质标准品(纯度均大于98%)以及C_32_正构烷烃标准品(纯度94.3%)均购于北京曼哈格生物科技有限公司; C_7_~C_40_正构烷烃混合标准溶液(1000 mg/L,上海安谱实验科技股份有限公司);乙腈(质谱纯,美国Thermo Fisher公司);无水硫酸镁(上海阿拉丁生化科技股份有限公司); GHP滤膜(0.22 μm,美国Waters公司)。

爽肤水、乳液、面膜、膏霜等样品来自网购平台及本地商场。

### 1.2 实验方法

#### 1.2.1 标准溶液的配制

称取15种防腐剂类过敏原物质标准品各100 mg(精确至0.1 mg),用乙腈分别定容至10 mL,配制成质量浓度为10 mg/mL的标准储备溶液。分别移取15种防腐剂类过敏原物质的标准储备溶液适量,用乙腈配制成100 μg/mL的混合标准中间液(-20 ℃避光保存)。临用时移取标准中间液,用乙腈逐级稀释配制得苯甲酸、甲基氯异噻唑啉酮、甲基异噻唑啉酮、5-溴-5-硝基-1,3-二噁烷质量浓度为0.01、0.05、0.1、0.5、1.0、2.0和5.0 μg/mL,其余11种组分质量浓度为0.01、0.05、0.1、0.2、0.5、1.0、1.5和2.0 μg/mL的系列混合标准工作溶液。

#### 1.2.2 样品处理

称取0.1 g(精确至0.001 g)样品于具塞比色管中,加入20 mL乙腈涡旋混匀,超声提取10 min后,用1 g(精确至0.01 g)无水硫酸镁除水,以10000 r/min离心5 min。取上清液过0.22 μm GHP滤膜,滤液备用。

### 1.3 仪器条件

#### 1.3.1 色谱条件

DB-5MS色谱柱 进样量1 μL;分流比15∶1;载气He(纯度>99.99%);载气流速1 mL/min;进样口温度280 ℃;程序升温:初始温度为50 ℃,保持3 min,以7 ℃/min的速率升温至100 ℃,保持2 min,再以10 ℃/min的速率升温至235 ℃,保持2 min,最后以20 ℃/min的速率升温至280 ℃。

DB-WAX色谱柱 进样口温度250 ℃;程序升温:初始温度为50 ℃,保持1 min,以50 ℃/min的速率升温至120 ℃,再以5 ℃/min的速率升温至195 ℃,保持3 min后,以20 ℃/min的速率升温至220 ℃,保持10 min,最后以15 ℃/min的速率升温至240 ℃,保持8 min。其他参数同DB-5MS色谱柱。

#### 1.3.2 质谱条件

离子源:EI源;离子源温度:230 ℃;传输线温度:280 ℃(DB-5MS柱)和250 ℃(DB-WAX柱);溶剂延迟时间:3.5 min(DB-5MS柱)和2.5 min(DB-WAX柱);数据采集模式:多反应监测模式(MRM)。15种防腐剂类过敏原物质的定量离子对、定性离子对及碰撞能量见表1。

**表1:** 15种防腐剂类过敏原物质的保留指数、定量离子对、定性离子对及碰撞能量

No.	Compound	CAS No.	DB-5MS				DB-WAX					
			RI	Ion pair (m/z)	Collision energy/V		RI	Ion pair (m/z)		Collision energy/V		
1	benzyl alcohol	100-51-6	1730	79.0>77.1^*^	15		1878		79.0>77.1^*^			12
				79.1>51.0	24				108.0>79.1			5
				79.0>53.1	21				79.1>51.0			21
2	benzoic acid	65-85-0	1855	105.0>77.1^*^	15		2621		122.0>105.1^*^			9
				105.0>51.1	27				105.0>77.1			12
				105.0>65.0	18				122.0>77.1			21
3	methylisothiazolinone	2682-20-4	1871	115.0>87.1^*^	12		2126		115.0>87.0^*^			9
				115.0>53.1	24				87.0>59.0			12
				115.0>59.1	24				115.0>53.0			24
4	isopropyl benzoate	939-48-0	1906	105.0>77.1^*^	15		1668		105.0>77.1^*^			12
				105.0>51.1	27				105.0>51.0			30
				105.0>75.1	24				77.0>51.0			12
5	bronidox	30007-47-7	1912	137.0>109.0^*^	9		/					
				137.0>58.0	15							
				137.0>107.0	15							
6	methylchloroisothiazolinone	26172-55-4	1922	149.0>121.0^*^	12		1976		149.0>121.0^*^			9
				149.0>85.0	21				149.0>85.0			18
				149.0>87.0	27				149.0>87.0			27
7	methylparaben	99-76-3	2151	121.0>93.1^*^	12		2939		152.0>121.1^*^			9
				121.0>65.1	21				121.0>65.1			18
				121.0>77.1	21				121.0>93.1			9
8	ethylparaben	120-47-8	2220	121.0>93.1^*^	12		2968		121.0>65.1^*^			18
				121.0>65.1	21				121.0>93.1			9
				121.0>77.1	24				138.0>121.1			12
9	isopropylparaben	4191-73-5	2251	121.0>93.1^*^	12		2943		138.0>121.1^*^			12
				121.0>65.1	21				121.0>93.0			12
				121.0>111.1	12				121.0>65.1			18
10	propylparaben	94-13-3	2321	121.0>65.1^*^	21		3060		138.0>121.1^*^			12
				121.0>93.1	12				121.0>65.1			18
				121.0>111.1	9				121.0>93.1			9
11	benzoic acid phenyl ester	93-99-2	2367	105.0>77.1^*^	15		2488		105.0>77.1^*^			12
				105.0>51.1	27				105.0>51.1			27
				105.0>79.1	12				77.0>51.0			12
12	isobutylparaben	4247-02-3	2380	121.0>93.1^*^	12		3097		138.0>121.1^*^			12
				121.0>65.1	21				121.0>65.1			18
				121.0>111.1	9				121.0>93.0			9
13	butylparaben	94-26-8	2425	121.0>65.1^*^	21		3171		138.0>121.1^*^			12
				121.0>93.1	12				121.0>65.1,			18
				121.0>111.1	9				121.0>93.1			9
14	phenylparaben	17696-62-7	2744	121.0>65.1^*^	21		/					
				121.0>93.1	12							
				121.0>111.1	12							
15	benzylparaben	94-18-8	2843	121.0>65.1^*^	21		/					
				121.0>93.1	12							
				121.0>111.1	12							

* Quantitative ion pair; /: cannot be detected.

### 1.4 保留指数的计算

分析物的保留时间因色谱柱类型等分析条件不同而不同,而保留指数(待测组分在相邻两个正构烷烃之间的相对位置)根据色谱柱类型保持不变,是广泛使用、国际公认的定性指针。分别在上述DB-5MS和DB-WAX仪器条件下采集C_7_~C_40_正构烷烃的出峰信息,由于仪器参数设置,如溶剂延迟时间、具体升温程序不同等,导致C_7_~C_40_正构烷烃在上述升温程序的采集范围内未全部出峰,无法准确判定每个具体正构烷烃的位置。此时通过采集单一C_32_烷烃,再准确鉴别上述程序中采集到的其他正构烷烃,确定每个正构烷烃的质谱信息,利用软件计算15种防腐剂类过敏原物质的保留指数。保留指数的计算公式:


(1) RI=100*Z+*100(*T_X_-T_Z_*)*/*(*T_Z+_*_1_*-T_Z_*)

其中,*Z*:目标峰相邻(之前)的正构烷烃的碳数;*T_X_*:目标化合物保留时间;*T_Z_*:目标峰相邻(之前)的正构烷烃的保留时间;*T_Z_*_+1_:目标峰相邻(之后)的正构烷烃的保留时间。

## 2 结果与讨论

### 2.1 色谱条件的优化

#### 2.1.1 分流比的选择

分析比较了分流比为50∶1、15∶1、不分流3种情况下同一实际加标样品在DB-5MS色谱柱上全扫描模式下的出峰情况,见附图S1~S3(详见 https://www.chrom-China.com)。当不分流进样时,15种防腐剂类过敏原物质的色谱峰均存在拖尾现象,且实际样品检测中引入较多杂质易污染离子源;当分流比为15∶1时,各物质均能较好分离,可以满足检测需求;当分流比为50∶1时,各物质分离度也可以满足检测需求,但较分流比为15∶1时灵敏度下降。考虑化妆品中各防腐剂含量及检测需求,选择分流比为15∶1。

#### 2.1.2 色谱柱的选择

目前使用GC、GC-MS分离检测多种组分的研究大多围绕对比多种不同色谱柱分离情况,选择单根最优色谱柱开展。实际应用时,存在检测上述15种防腐剂类过敏原物质的同时需检测其他物质的情况,单一柱系统可能不能满足应用需求。选用极性相差较大的双色谱柱建立研究,可以采集到不同极性段的物质,弥补单色谱柱检测中存在的不足,同时也可以在非定向筛查时扩大物质的检测范围,筛查出更多极性范围相差较大的物质,结合保留指数定性,可以提供更丰富的物质信息,扩大实际应用范围。

研究选取DB-5MS和DB-WAX色谱柱搭建GC-MS双柱系统。其中,DB-5MS色谱柱为非极性色谱柱,对非极性、弱极性以及中等极性的成分都有较好的分离度。同时,其信噪比高,灵敏度高,耐高温,可以较好地分离高沸点物质,本研究中的15种待测组分均被检测到,但其可能不能很好地适用于其他极性成分的检测。DB-WAX柱为强极性色谱柱,适用于分离强极性、低沸点成分,为极性物质提供了对称的峰形,提高极性物质的响应度。但DB-WAX色谱柱最高柱温250 ℃,不耐高温,无法检测高沸点、不易挥发的组分,如5-溴-5-硝基-1,3-二噁烷、4-羟基苯甲酸苯酯、4-羟基苯甲酸苄酯,且随着温度升高,DB-WAX色谱柱会发生基线漂移现象。对于部分极性较强的物质,DB-WAX的保留作用强,可能会存在色谱峰拖尾的情况。本研究通过DB-WAX色谱柱,可以检测到12种待测组分,但甲基异噻唑啉酮及苯甲酸酯类防腐剂在DB-WAX条件下的检出限和定量限均低于DB-5MS色谱柱。采用DB-5MS和DB-WAX色谱柱时15种防腐剂类过敏原物质的总离子流色谱图见图1。

**图1:**
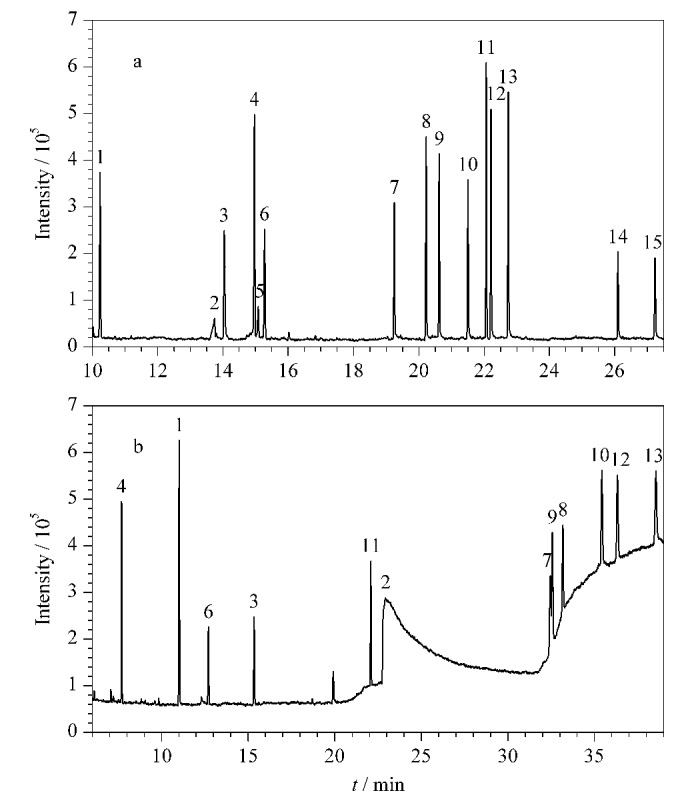
15种防腐剂类过敏原物质分别采用(a) DB-5MS和(b) DB-WAX色谱柱分离检测时的总离子流色谱图

### 2.2 质谱条件的优化

根据GC-MS/MS母离子和子离子一一对应的多反应监测模式,通过设定多个时间段和扫描通道同时分析多种防腐剂类过敏原物质成分,先通过GC对其分离和全扫描,确定防腐剂成分的出峰时间和一级碎片离子,选择离子强度高的一级碎片作为母离子,应用电子轰击电离模式对母离子在不同碰撞能量下进行二次电离,选择信号较强的二级碎片离子作为子离子,以产生信号强度最强的碰撞能量作为最终优化碰撞能量,优化后的条件见表1。

### 2.3 定性方式的优化

区别于简单的保留时间定性,本文利用保留指数对15种防腐剂类过敏原物质进行定性。保留指数定性方式误差小,应用范围广。在非定向筛查时,利用保留指数参数,也可以在无标准品或不使用标准品的情况下,仅通过分析一次正构烷烃,即可预估目标物的保留时间,可以为非定向筛查提供思路,具有重要应用价值。在DB-5MS和DB-WAX色谱柱上首先确定每个正构烷烃的保留时间,每个正构烷烃的保留指数对应100倍正构烷烃的碳数。根据15种防腐剂类过敏原物质的保留时间及峰前后正构烷烃的保留时间计算其保留指数,即15种防腐剂类过敏原物质的所对应峰前后相邻正烷烃的相对保留时间。在DB-5MS、DB-WAX条件下测得的C_32_和C_7_~C_40_正构烷烃的出峰情况如图2、3所示,15种防腐剂类过敏原物质的保留指数详见表1。由于苯甲酸在DB-WAX柱上拖尾现象严重,随浓度升高轻微峰前移,故苯甲酸在DB-WAX柱上的保留指数定性窗口较宽,具体15种防腐剂类过敏原物质在DB-5MS、DB-WAX色谱柱条件下保留指数的定性窗口如附表S1所示。

**图2:**
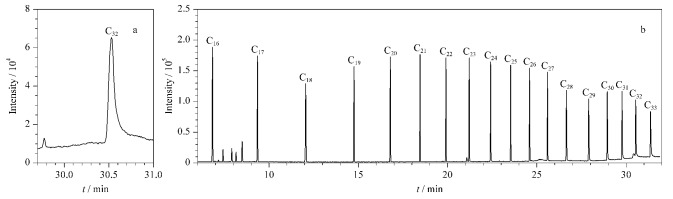
(a)C_32_和(b)C_16_~C_33_烷烃在DB-5MS条件下的提取离子流色谱图

**图3:**
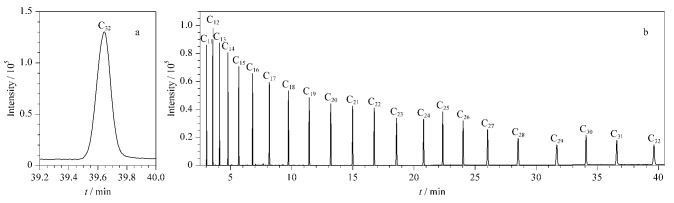
(a)C_32_和(b)C_11_~C_32_烷烃在DB-WAX条件下的提取离子流色谱图

### 2.4 样品提取方式的优化

#### 2.4.1 样品提取溶剂的选择

本方法对比了化妆品中15种防腐剂类过敏原物质在甲醇和乙腈两种溶剂中的提取效果,虽然乙腈提取可能引入更多的杂质,但对苯甲酸等组分的提取效果优于甲醇,且加标回收率基本满足《分析方法验证指导原则》的要求,因此选择乙腈作为提取溶剂。样品溶液含水会使灯丝氧化,降低灯丝寿命,因此选择无水MgSO_4_作为脱水试剂。前处理方法简单方便,满足检测需求。

#### 2.4.2 样品提取溶剂用量的选择

研究对比了10 mL和20 mL两种提取溶剂用量下实际样品中15种防腐剂类过敏原物质的出峰情况。10 mL乙腈提取时部分样品存在检测器饱和现象,降低仪器寿命,20 mL乙腈提取时则几乎不存在检测器饱和的问题,且选取的爽肤水、乳液、面膜、膏霜4种基质均为免洗型产品,依据《技术规范》中对这15种防腐剂类过敏原物质的禁、限量要求,最大允许使用浓度均落在方法线性范围内,满足监管要求,因此选择乙腈提取溶剂的体积为20 mL。

#### 2.4.3 净化方式的选择

由于15种防腐剂类过敏原物质的极性相差较大,因此没有选择固相萃取技术。直接超声提取的预实验发现,爽肤水、乳液、面膜、面霜4种常见基质在DB-5MS和DB-WAX色谱条件下均存在基质效应(ME),通过HLB小柱进行净化处理,对比净化前后的实验结果,各基质加标样品的基质效应降低,但苯甲酸、甲基异噻唑啉酮等组分回收率也降低,不能满足实验要求。故最终仍选择直接超声提取,不经HLB小柱净化,通过基质匹配标准曲线定量降低基质效应的影响。

#### 2.4.4 超声时间的选择

超声提取可以增大待测组分的提取效率,但同时加快了易挥发组分的流失。实验分别对涡旋混匀(肉眼观察混匀后既可)、涡旋混匀5 min、涡旋混匀(肉眼观察混匀后既可)后超声提取一定时间(5、10、15、20、25 min)进行了考察。超声10 min时,待测组分的回收率都相对较高,如图4所示。因此,实际样品提取选择超声10 min。

**图4:**
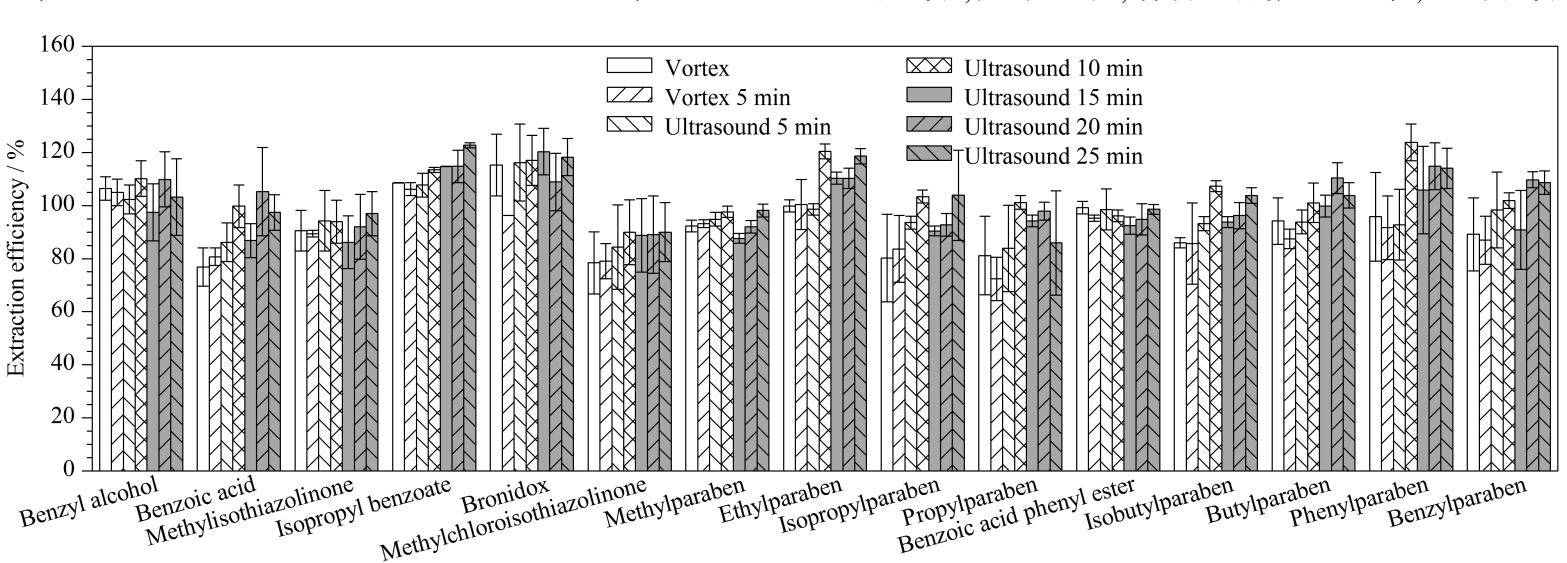
不同提取方式和时间条件下15种防腐剂类过敏原物质的提取效率(*n*=6)

### 2.5 方法学评价

#### 2.5.1 基质效应的考察

基质效应会影响分析结果的准确性,可以用基质匹配标准曲线斜率和溶剂标准曲线斜率的比值评价基质效应。按照1.2.1节配制系列混合标准溶液,在确定的最佳仪器分析条件下进样测定,以目标组分的峰面积对相应的质量浓度绘制溶剂标准曲线;向空白样品中添加15种防腐剂过敏原混合标准溶液,同法获得基质匹配标准曲线。ME越接近100%,说明基质效应越小。DB-5MS色谱柱条件下基质效应的范围为63.6%~149.9%,DB-WAX色谱柱条件下基质效应的范围为88.0%~114.4%。结果表明,15种化合物在4种基质中存在基质抑制和增强效应,且DB-WAX色谱柱的基质效应弱于DB-5MS色谱柱(详见附表S2~S3)。为降低基质效应的影响,本实验采用基质匹配标准曲线法进行定量分析。

#### 2.5.2 线性范围与检出限

在最优测定条件和提取条件下,对该方法的线性范围、检出限(LOD)和定量限(LOQ)进行验证,以定量离子信噪比(*S/N*)大于3确定LOD,以*S/N*大于10确定LOQ。15种防腐剂类过敏原物质在DB-5MS、DB-WAX色谱柱条件下的线性范围、相关系数(*r*^2^)、检出限、定量限如表2所示。经两次检测,DB-5MS色谱柱可以同时检测15种防腐剂类过敏原物质,方法的检出限为0.02~0.2 mg/kg; DB-WAX色谱柱可以检测12种防腐剂,方法的检出限为0.01~20 mg/kg。在各自的线性范围内15种防腐剂类过敏原物质的线性关系良好,相关系数均大于0.99。与文献[25]相比,本实验在DB-5MS条件下获得15种组分的检出限和定量限值更低,检测的灵敏度更高。DB-WAX为强极性色谱柱,对极性组分的分离效果好,但其不耐高温,不适用于高沸点、不易挥发的组分,如5-溴-5-硝基-1,3-二噁烷、4-羟基苯甲酸苯酯、4-羟基苯甲酸苄酯;对于部分极性较强的组分,如苯甲酸,容易造成拖尾现象,对峰积分造成了极大程度的影响。杨明等^[26]^的研究也表明苯甲酸在极性色谱柱上易拖尾,因此造成苯甲酸的检出限和定量限值较高,但甲基异噻唑啉酮以及苯甲酸酯类防腐剂在DB-WAX条件下的检出限和定量限低于DB-5MS色谱柱,定量检测时检出限相对更低。

**表2:** 15种防腐剂类过敏原物质的线性范围、相关系数、检出限与定量限

Compound	DB-5MS					DB-WAX				
	LOD/ (mg/kg)	LOQ/ (mg/kg)	Linear range/ (mg/L)	r^2^		LOD/ (mg/kg)	LOQ/ (mg/kg)	Linear range/ (mg/L)		r^2^
Benzyl alcohol	0.1	0.2	0.01-2	0.9941		0.04	0.2	0.01-2		0.9994
Benzoic acid	0.2	0.4	0.01-2	0.9940		20	40	0.5-5		0.9992
Methylisothiazolinone	0.1	0.2	0.01-2	0.9934		0.02	0.2	0.01-5		0.9994
Isopropyl benzoate	0.04	1	0.01-2	0.9952		0.01	0.02	0.01-2		0.9993
Bronidox	0.2	0.4	0.01-5	0.9948		/	/	/		/
Methylchloroisothiazolinone	0.04	0.1	0.01-2	0.9951		0.04	0.2	0.01-5		0.9994
Methylparaben	0.04	0.1	0.01-2	0.9940		0.04	0.2	0.01-2		0.9997
Ethylparaben	0.04	0.1	0.01-2	0.9902		0.04	0.2	0.01-2		0.9997
Isopropylparaben	0.04	0.1	0.01-2	0.9903		0.04	0.2	0.01-2		0.9997
Propylparaben	0.04	0.1	0.01-2	0.9923		0.04	0.1	0.01-2		0.9997
Benzoic acid phenyl ester	0.04	0.1	0.01-2	0.9912		0.02	0.04	0.01-2		0.9996
Isobutylparaben	0.04	0.1	0.01-2	0.9947		0.04	0.1	0.01-2		0.9995
Butylparaben	0.04	0.1	0.01-2	0.9932		0.04	0.2	0.01-2		0.9998
Phenylparaben	0.02	0.04	0.01-2	0.9903		/	/	/		/
Benzylparaben	0.04	0.1	0.01-2	0.9910		/	/	/		/

#### 2.5.3 回收率和精密度

向爽肤水、乳液、面膜和膏霜4种基质的空白样品中添加3个不同水平的混合标准溶液,进行方法回收率验证。DB-5MS条件下,各防腐剂类过敏原物质的添加水平均为0.02、0.2、1 mg/L; DB-WAX条件下,苯甲酸的添加水平为0.5、1.5、4 mg/L,其他防腐剂类过敏原物质的添加水平为0.02、0.2、1 mg/L。结果表明,各防腐剂类过敏原物质在DB-5MS条件下和在DB-WAX条件下回收率在70.1%~129.8%范围内,相对标准偏差(RSD)均小于15%,详见附表S4~S5。

#### 2.5.4 保留指数的精密度

为进一步考察混合标准溶液保留指数的精密度,在相同条件下测定混合标准溶液和正构烷烃,确定15种防腐剂类过敏原物质的保留指数,各组分保留指数的相对标准偏差小于0.0035%(*n*=6),表明使用正构烷烃对待测物定性时相对误差小,可信度高。

### 2.6 实际样品分析

按所建立的方法对爽肤水、乳液、面膜和膏霜4种基质各20批实际样品进行分析,由于苯甲酸在DB-WAX色谱柱上拖尾现象严重,检出限高于DB-5MS色谱柱,导致产品中苯甲酸在DB-5MS条件下的检出率高于DB-WAX柱;甲基异噻唑啉酮在DB-WAX色谱柱条件下的检出限为0.02 mg/kg,低于在DB-5MS色谱柱条件下的检出限0.1 mg/kg,导致甲基异噻唑啉酮在DB-WAX的检出率为8.8%,略高于DB-5MS条件下的检出率7.5%。80批实际样品中有2批检测出了禁用成分4-羟基苯甲酸异丙酯,如表3所示。

**表3:** 80批实际样品中15种防腐剂类过敏原物质的检出率

Compound	DB-5MS/%	DB-WAX/%
Benzyl alcohol	42.5	42.5
Benzoic acid	33.8	22.5
Methylisothiazolinone	7.5	8.8
Isopropyl benzoate	12.5	12.5
Bronidox	17.5	/
Methylchloroisothiazolinone	6.3	6.3
Methylparaben	53.8	53.8
Ethylparaben	12.5	12.5
Isopropylparaben	2.5	2.5
Propylparaben	18.8	18.8
Benzoic acid phenyl ester	23.8	23.8
Isobutylparaben	not detectable	not detectable
Butylparaben	1.3	1.3
Phenylparaben	not detectable	/
Benzylparaben	not detectable	/

## 3 结论

使用GC-MS技术建立了基于“两柱保留指数、一个质谱匹配度”的化妆品中15种防腐剂类过敏原物质的精确鉴定方法,采用MRM的采集方式提高了检测方法的灵敏度。与大多数防腐剂类过敏原物质检测方法相比,该法灵敏度、准确性高,有效避免了假阳性、假阴性的检测结果,适用于多种基质化妆品中多种防腐剂的同时测定。使用保留指数进行定性,以期为非定向筛查提供思路,可以对实验条件下测得的保留指数进行匹配,初步判断样品中是否含有15种防腐剂类过敏原物质。同时双色谱柱的使用可以在非定向筛查时结合实际应用进一步扩展,囊括更多未知组分,扩大应用范围。
